# Anaemia in Ruminants Caused by Plant Consumption

**DOI:** 10.3390/ani12182373

**Published:** 2022-09-11

**Authors:** Héctor Ruiz, Delia Lacasta, Juan José Ramos, Hélder Quintas, Marta Ruiz de Arcaute, María Ángeles Ramo, Sergio Villanueva-Saz, Luis Miguel Ferrer

**Affiliations:** 1 Animal Pathology Department, Instituto Agroalimentario de Aragón-IA2, Universidad de Zaragoza-CITA, Veterinary Faculty of Zaragoza C/Miguel Servet 177, 50013 Zaragoza, Spain; 2 Mountain Research Centre (CIMO), School of Agriculture, Polytechnic Institute of Bragança (IPB), Campus de Santa Apolónia, 5300-253 Bragança, Portugal

**Keywords:** plants poisoning, anaemia, ruminants, onion toxicosis, bracken fern poisoning, giant fennel poisoning, sweet clover poisoning, *Brassicaceae* poisoning

## Abstract

**Simple Summary:**

Plant poisoning is an underdiagnosed condition affecting livestock, and is especially important in ruminants. It is necessary for the veterinary practitioner to consider these aetiologic agents when confronting anaemia in ruminants. Although many of these plants are well-known by veterinarians and farmers, their toxic effects remain unnoticed by many of them. Due to the poisonous clinical signs, anaemia caused by plant consumption should be divided into two groups: haemorrhagic disorders and haemolytic disorders. The knowledge of their presence and possible consumption of the plant, together with the clinical signs, will lead to early diagnosis and appropriate treatment of the affected animals. The objective of this review is to show and organise the plants that most frequently produce anaemia in ruminants based on the two groups previously described.

**Abstract:**

Plant toxicology has affected animals throughout evolution. Plants have adapted themselves to the environment. This adaptation has led to the development of defensive strategies to avoid being consumed. Plants have several chemical compounds, which can cause deleterious effects on people or animals that consume them, causing a wide variety of clinical signs. Plants from various latitudes, both cultivated for human and animal feeding or decorative purpose and even wild growth plants are able to generate anaemia in ruminants. Coumarins or ptaquiloside predispose bleeding and haemorrhages, causing a haemorrhagic disease in affected animals. In this group, some important fodder plants, such sweet clover (Genus *Melilotus* spp.), or other weeds distributed worldwide, such as bracken fern (*Pteridium aquilinum*) of giant fennel (*Ferula communis*), are included. On the other hand, sulfur-containing chemicals (e.g., n-propyl disulfate and S-propyl cysteine sulfoxides (SMCOs)) may cause severe direct damage to the erythrocyte and their membrane, leading to their destruction and causing haemolytic anaemia in the animal. This review presents the most frequent intoxication by plants causing anaemia in ruminants. Toxic compounds, clinical signs, diagnosis and possible treatments are also presented.

## 1. Introduction

Plant toxicology is an issue that has been closely related to humanity and animals. In its adaptation to the environment, plants have successfully developed defensive strategies, either chemical or physical, to avoid being consumed by animals [1]. Thereupon, several compounds such as alkaloids, glycosides and oxalates, present in plants or their extracts, have given them pharmacological properties used to treat certain diseases in humans and animals since ancient times [1,2]. However, due to factors related to the type of plant, animal and/or management system, they can also be harmful, and the aphorism “Only doses makes a poison”, credited to Paracelsus, can find its maximum expression in plants toxicology [1].

Poisonous plants cause significant economic losses to the livestock industries worldwide, including direct and indirect costs [2,3]. Although the real impact is not well noticed, it is considered to cause more considerable economic losses in meat farms than viruses, parasites and bacteria together [4]. Data from the Centre National d’Informations Toxicologiques Vétérinaires (CNITV) in Lyon, France, showed 11% of domestic animal poisoning to be attributable to plants [5], while the Poison Control Centre of Milan showed that plant poisoning accounted for 5.5% [4]. In these studies, all domestic animals were included. Nevertheless, its impact on herbivores, especially ruminants, is of particular concern [6], especially those reared in extensive o semi-intensive conditions, which consume plants while grazing. These plants may contain anti-nutritional and toxic compounds responsible for a wide range of deleterious effects [3,7]. It must be taken into account that plant poisoning can also occur in intensive management systems, although to a lesser extent, so care must be taken in formulating the feed to avoid accidental poisoning [8]. In cattle and small ruminants, plants are the second most common class of toxicants after pesticides [9].

In the clinical approach to anaemia in ruminants, several causes must be ruled out: internal or external parasites (e.g., digestive parasites, fleas, lice), infectious (e.g., babesiosis, anaplasmosis, leptospirosis, clostridia toxins), metabolic (e.g., vitamin or mineral deficiencies, concurrent chronic diseases or neoplasias), haemorrhagic (e.g., trauma, dystocia, abomasal ulcers, vena cava thrombosis, haemorrhagic bowel syndrome), chemical poisoning (e.g., heavy metals, drugs, rodenticides) or less frequently immune-mediated anaemia (e.g., feeding new-born lambs (*Ovis aries*) with cows’ (*Bos Taurus*) colostrum) and diseases of bone marrow [10,11]. Usually, for the veterinary practitioner, it is difficult to diagnose and include anaemia caused by the consumption of plants in this list.

Plants from various latitudes, cultivated for human and animal feeding, decorative purpose or spontaneous growth, are able to generate anaemia in ruminants. Some of these plants have toxic compounds such as alkaloids, glucosinolates, glycosides, oxalates and coumarins inherent to their condition. However, the harmful substances can accumulate in the plant in certain conditions, such as severe droughts, high-speed growth or during flowering [9,12]. In order to include these poisoning in the differential diagnosis when confronting anaemia in ruminants, it is crucial to classify toxic plants or families according to the clinical signs that the plant’s toxic compounds can produce. Attending to anaemia disorders by consumption of plants, it is important to point out two groups: plants causing haemorrhagic disorders and plants causing haemolytic anaemia [12]. 

## 2. Haemorrhagic Disorders

These disorders, often fatal to ruminants, are mostly caused by plants containing anticoagulants in variable proportions. The amount of anticoagulant compounds present in the plant will determine the severity of the clinical signs. The consumption of these plants is avoided by the ruminants. However, in times of food scarcity caused by extreme droughts, excessive grazing or oversizing pastures, animals can eat them [7,12].

Some plants included in this group contain high amounts of coumarin group compounds [13]. Coumarins’ mechanism of action lies in their specific action as a competitive antagonist of vitamin K. This action prevents the production of prothrombin and several other coagulation factors, inhibiting the coagulation of blood [14]. This characteristic action has allowed using these compounds as a pharmacological treatment for systemic embolism and for preventing venous thromboembolism [15,16]. On the contrary, coumarins have also been used as rodenticides due to the same effect [17].

**Figure 1:**
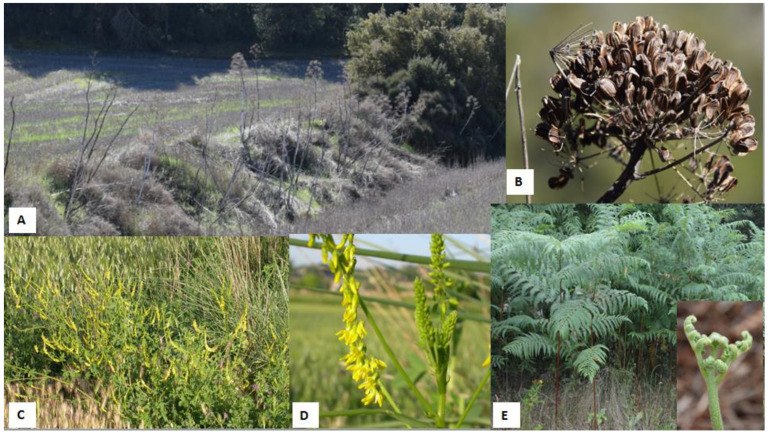
Some examples of plants associated with haemorraghic disorders. (**A**) Giant fennel (*Ferulla communis*) on an embankment. (**B**) General aspect of fruits of giant fennel. (**C**) Sweet clover (*Melilotus officinalis*) grown on the edge of a wheat field. (**D**) Flower of *M. officinalis*. (**E**) General aspect and fronds of bracken fern (*Pteridium aquilinum*). The small picture shows a “fiddlehead” sprouting.

In ruminants, haemorrhagic disorders caused by coumarins occur after the consumption of mainly two types of plants: giant fennel (*Ferula communis*) and sweet clover (*Melilotus officinalis*) [7,12]. Coumarins and natural derivatives could be found in some other plants such as tonka bean (*Dipteryx odorata*), vanilla grass (*Anthoxanthum odoratum*), woodruff (*Galium odoratum*), mullein (*Verbascum* spp.) and sweet grass (*Hierochloe odorata*) [12,18] (Figure 1).

In addition, another important plant, the bracken fern (*Pteridium aquilinum*), must also be included in this group. The haemorrhagic disorder caused by bracken fern is not mediated by coumarins. In this case, the anaemia is due to platelet depletion [12].

### 2.1. Giant Fennel (Ferula communis)

It is a perennial, herbaceous and robust weed (up to several meters in height) belonging to the *Apiaceae* family, which is widespread in Central Asia, the circum-Mediterranean area and the Macaronesian Region [19,20,21]. The plant has alternate, petiolate leaves with linear lobes. The flowers have yellow petals and no sepals, while the fruit is elliptical, about 15 mm long and dorsally compressed. The whole plant is toxic, both fresh and hay [12,22]. It is normal to see the plant on verges and embankments in dry and rocky soils.

In naturally grazing conditions, outbreaks occur more frequently after droughts or food shortages [12]. Some studies have determined that in lambs, daily consumption of 2.5–5 g of plant per kilogram of body weight can cause intoxication [23].

It is considered that there are two chemotypes which cannot be distinguished botanically despite one being highly toxic while the other one being non-toxic [24,25]. The toxic variety is considered to be responsible for a lethal haemorrhagic syndrome which is also called ferulosis [22]. This intoxication can affect sheep (*Ovis aries*), goats (*Capra aegagrus hircus*), cattle (*Bos Taurus*) and horses (*Equus caballus*) [23,26,27].

Some chemical investigations have reported important differences in the chemical compounds present in both chemotypes. While the non-toxic chemotype mostly presents daucane esters or drimane ethers as the main constituents, the poisonous chemotype mainly contains prenylated coumarins. Being the ferulenol (a 4-hydroxycoumarin derivative) and ferprenin, a pyrane (3,2-c) coumarin derivative, the most toxic compounds of the plant. In addition, other hidroxicoumarins can be found in the toxic variety [12,22,24,28]. These toxic compounds are in the plant’s parts (leaf, root, flower and stem) [29]. Both ferulenol and ferprenin are specific inhibitors of the VKORC1 enzyme expressed in the liver of all mammals [22,29]. This enzyme catalyses the vitamin K 2,3-epoxide reductase activity, being essential for recycling vitamin K. Inhibition of VKORC1 by these derivative coumarins limits the amount of vitamin K hydroquinone (KH2), resulting in a block of the clotting factors II, VII, IX and X activation, leading to uncontrollable bleeding episodes [30]. Both ferulenol and ferprenin can inhibit VKORC1 activity in a non-competitive way, as observed with warfarin. Although all mammals are susceptible, there is variability in susceptibility between species, with cattle being the most susceptible to this intoxication [22].

This haemorrhagic syndrome is evidenced by the presence of bleeding from the nostrils or even blood in stools. Pale mucous membranes, muscle weakness, arrhythmias, shallow breathing and even death, may be associated with this syndrome [12,26,27]. Clinical signs appear within 24–48 h after consuming the plant [12].

Diagnosis is based on clinical signs, basically in the presence of haemorrhages. An increase in clotting time may be observed. Profuse haemorrhages in mucous membranes, subcutaneous tissue and even in the thoracic and abdominal cavities can be appreciated at necropsy [12].

There is no specific treatment for this disturbance. However, it is considered that in goats and cattle, a therapy based on intramuscular injection of vitamin K1 (1–5 mg/kg BW, 1 to 7 days) is effective [12]. It can also be treated with supportive treatment with liver protectors. A blood transfusion should be considered in severe cases [12,27].

Ferulosis appears to be limited to a few areas of the western Mediterranean region, although *F. communis* is widely distributed. This would be due to a different distribution of both chemotypes [29].

### 2.2. Bracken Fern (Pteridium aquilinum)

Bracken fern is a widely distributed plant belonging to the family *Dennstaedtiaceae* [12]. It is a herbaceous, perennial plant considered to be one of the most successful invasive plant species [31,32]. The plant is also named Eagle fern and is characterised by an extensive, repeatedly branched underground rhizome system and large fronds 15(30)–180(440) cm [32,33].

The rhizome is dark, thick, deep and rich in starch [12] which has sometimes been crushed into flour of very poor quality [31]. Early every spring, the so-called “leafs” sprout from the rhizome and are known as “fiddleheads”. These fiddleheads are curled and covered with silver grey hair [34]. Fronds are very divided, tri-pinnate, but variable, ranging from two- to four-pinnate and supported by a vertical, long, hard and blackish base stem [12,32]. Fronds are shaped similar to a triangle [31]. During late autumn, especially in northern areas, fronds dry out, and the rhizomes remain buried, waiting for the spring [31,33].

*P. aquilinum*, like other ferns, has neither flowers nor fruits. However, the plant has fertile fronds similar to sterile ones in which sporangia are located beneath the outer margins of the leaflets [31,34]. The clusters of spores form nearly unbroken lines along the edges of leaflets, and up to 30 million spores may be produced by a single frond [34].

This fern is present in all continents except Antarctica [35], preferably in the humid mountainous regions of temperate climate, with medium to high annual rainfall. It also prefers acidic, shaded and well-drained soils [12,31,36].

The bracken fern has spread enormously since the 1980s, closely linked to the abandonment of pastures and rural areas [37]. It is a ruderal plant that, in natural conditions, is developed in humid and shady ravines [12]. However, it is increasingly common to find it in bordering areas after fire [38], after persistent frosts [36] and in degraded forests, predominantly of pine and oak [12]. These situations generate that bracken tends to form dense and uniform grazing areas with low feeding value and potential toxicosis risk for livestock [39,40]. For this reason, bracken fern is considered among the world’s worst weeds [41].

The whole plant is toxic and has several toxic compounds, such as pruinose (a cyanogenic glucoside), ptachiloside (PTQ) (a carcinogen) and thiaminases (enzymes) [31,36]. In monogastric animals, the most frequent condition is a neurological disease caused by thiaminases which is less common in ruminants due to the ability of ruminal bacteria to synthesise thiamine [31]. On the contrary, ruminant clinical signs are mainly caused by ptachiloside action, causing both acute and chronic poisonings [12,36].

PTQ is a chemical compound that was discovered in 1983 [42,43]. A radiomimetic action has been associated with this compound, causing severe affection on the bone marrow in a similar way produced by ionising radiation. This action causes a decreased production of platelets and neutropenia, both secondary to bone marrow injury [36,44,45]. At least three different disorders have been directly associated with the toxic effect of this compound: acute haemorrhagic syndrome, bovine enzootic haematuria (BHE) and upper alimentary tract carcinomas [36].

Acute haemorrhagic syndrome or haemorrhagic diathesis is associated with the acute effect of PTQ [36,46]. This disease affects primarily young cattle, although sheep, buffaloes (*Bubalus bubalis*) and adult cattle can also be affected [12,31]. Hunger or lack of proper quality fodder triggers this poisoning. However, long periods of several weeks or even months consuming significant amounts of bracken fern (up to 10 g/kg BW) are necessary to develop the clinical signs [47]. The first clinical signs to appear are hyperthermia, inappetence, weakness and even prostration [36,48,49]. At the same time, pale and multifocal petechial haemorrhages in mucous membranes may be observed [12,49]. Epistaxis, melena and uncontrollable bleeding after skin puncture or insect bites are also commonly observed [49].

The clinical signs of the haemorrhagic syndrome, the haematological results (severe thrombocytopenia), and the access to bracken fern in the pastures guide the diagnosis [12,36,48]. Without treatment, almost all animals affected by an acute crisis die between 1 to 7 days after the onset of the clinical signs [48,49].

At necropsy, one of the most common findings is the presence of disseminative bleeding, including petechial to suffusive haemorrhages in mucous membranes, subcutaneous tissue or even haemorrhages in muscle. In the serous surface of gastric tract organs as well as in the heart, pericardium and pleura haemorrhages may also be observed [12,48,49]. Haemorrhagic content may be observed in the small and large intestinal lumen [49].

Histological examination revealed a severe reduction in all three lineages of hematopoietic cells of the bone marrow. Disseminated haemorrhages can also be observed in different organs and secondary disease-associated lesions [36,48].

There is no specific treatment. Despite receiving symptomatic treatment, many of the affected animals die [12,49]. Some authors consider that rapid implementation of the treatment is effective [36]. However, no good prognosis is observed in clinical cases [48,49]. A blood transfusion and fluid therapy are recommended. Broad-spectrum antibiotics, anti-inflammatories and vitamins can also be considered, although they have limited effect [36,49].

### 2.3. Sweet Clover (Genus melilotus)

The genus *Melilotus* belongs to the *Fabaceae* family native to Eurasia and has become naturalised worldwide [50]. It is an aromatic forage legume containing 19 species. Three of these species (*M*. *albus*, *M. officinalis* and *M. indicus*) are being commonly cultivated due to their great value as feed, medicinal or honey properties, and even as a wildlife habitat enhancer [51,52,53]. The genus name is due to the plant’s sweet smell [54]. During the past years, *Melilotus* has received much attention due to its tolerance to extreme environmental conditions. In addition, it has an excellent nitrogen fixation rate, being considered a good alternative in crop rotation and as a crop fertiliser [54,55]. The plant is even recommended as phytoremediation of soils contaminated with heavy metals and hydrocarbons [56].

It is an annual to biennial plant with strong taproot, trifoliate leaves and 30- to 70-flowered racemes of white (*M. albus*) or yellow (*M. officinalis* and *M. indicus*) flowers. It is an erect, freely branched plant that can reach near to 2 m in height [51,57,58]. The seed is black to dark-grey in colour, indehiscent, 2–4 mm, ovate, compressed but thick and has transverse ridges. This genus grows in partial shade or full sun areas and calcareous or loamy soils with low fertility [51,57]. In addition, *Melilotus* can also grow along paths, in ditches, and in fallows [59].

Sweet clover has traditionally been used in conventional medicine due to its multiple effects: antimicrobial, dermatological regeneration, hypotensive, antioxidant and even a neuro and hepatoprotective effect [58,60,61]. The wide variety of plant effects is based on the plant’s multiple chemical compounds, such as phenolic acids, steroids, saponins, coumarins, volatile oils and glycosides [59,60,61]. Regarding livestock, coumarins have received much attention due to their anticoagulant action. In 1921, Schofield described fatal bleeding in cattle when fed with sweet clover forage [62]. However, it was observed that the disease happened when the forage was poorly conservated. When the forage is mouldy, the coumarin is broken down by fungi, usually associated with the genus *Aspergillus* or *Penicillium* [59], and a new compound known as dicoumarol is generated [62,63,64,65].

When improper curing and harvesting occur, a fungus grows in *Melilotus* forage, most commonly in silage than hay. This growth generates a dimerisation of coumarin in dicoumarol which is able to act as a potent anticoagulant, very similar to those used as rodenticides (warfarin) [61,66]. This compound is an anti-vitamin K anticoagulant acting as a competitive antagonist of vitamin K. This biological action avoids the production of prothrombin and other coagulation factors, preventing blood coagulation [14]. Cattle seem more susceptible to the poisoning while sheep are apparently resistant [59].

Clinical signs develop after several weeks of consumption. Chronic consumption of concentration of 10 to 20 mg of dicoumarol per kg of hay is sufficient to cause poisoning in cattle in 100 days. When the dose is from 60 to 70 mg/kg of hay, poisoning can happen in just in 15 days [66]. It is common to observe some lame animals as the first clinical sign due to muscle haemorrhages [59,67]. Sometimes sudden death caused by a massive internal haemorrhage may also be the first sign [64]. Other common symptoms are epistaxis, haematuria, depression, reluctance to move, subcutaneous haematomas, and pale mucous membranes. When blood loss is severe, haemorrhagic anaemia signs appear, showing tachypnea, tachycardia and debility [59,64,65,66,67].

Diagnosis is based on clinical signs, although it is necessary to rely on complementary analysis. A haematological test will reveal severe anaemia with increased clotting and prothrombin times [59,64]. Despite its complexity, the isolation and quantification of dicoumarol in forage are also recommended [64,67]. At the herd level, the diagnosis of mouldy sweet clover poisoning in one animal suggests the presence of the disease in more animals [64].

At necropsy, diffuse haemorrhages in almost any organ or tissue can be identified. Even the thorax and abdominal cavity may be full of blood [64,67]. Microscopically, diffuse centrilobular necrosis of the liver is observed. Multifocal haemorrhages may be observed in the lung, intestine, kidney, urinary bladder sections, etc. Cardiac and skeletal muscle contain multifocal areas of myofiber degeneration and necrosis associated with haemorrhages [67].

There is no specific treatment for the disease. It is mandatory to stop feeding animals with suspected forage. However, new cases can appear after that [64]. A whole blood transfusion (10 mL/kg body weight) is recommended in severely affected animals [68]. A massive dose of 2000 mg of vitamin K1 should restore prothrombin time within 24 h [64,66,68].

Fortunately, varieties with low coumarin content are being developed, making it difficult to reach the toxic dose despite poorly conservation [53]. In addition, forage conservation technologies are improving, and therefore the preservation of forage has improved.

## 3. Haemolytic Disorders

Hemolysis can be produced by a wide variety of etiologic agents such as parasites (*Babesia* spp., *Theileria* spp. and *Anaplasma* spp.), bacteria (*Clostridium perfringens* type D or *Leptospira* spp.), poisoning by heavy metals (copper (Cu), lead (Pb), zinc (Zn)), idiopathic causes and even by plant consumption [10]. These plants contain some compounds capable of altering the metabolism of the erythrocytes and damaging their membrane producing haemolysis [12,69]. The excess of free haemoglobin in plasma is eliminated through the kidneys, leading to haemoglobinuria, giving a characteristic dark red colour to the urine.

Coincidentally, most plants with the highest risk of producing haemolytic anaemia in ruminants are widely cultivated throughout the world for human feeding. These plants can be included in the genus *Allium* spp. and the family *Brassicaceae* [12]. In addition, some other wild brassicas such as charlock mustard (*Sinapis arvensis*), wild radish (*Raphanus raphanistrum*), purple mistress (*Moricandia arvensis*) and black mustard (*Brassica nigra*) can cause haemolytic disorders in animals as well as some other plants belonging to other families such as tanner-grass (*Brachiaria radicans*), *Ditaxis desertorum*, *Indigofera suffruticosa* and the pink morning glory (*Ipomoea carnea*) [10,12,70,71].

### 3.1. Allium spp. pl.

**Figure 2:**
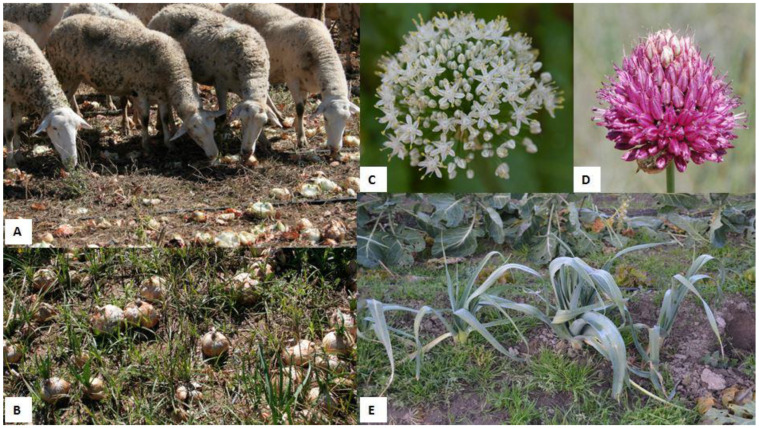
(**A**) A sheep herd grazing on an onion (*Allium cepa*) field. (**B**) Onion (*A. cepa*) field ready to harvest. (**C**) Characteristic umbel of white flowers of an onion (*A. cepa*). (**D**) Umbel of pink flowers of a round-headed leek (*A. sphaerocephalon*). (**E**) Leeks (*A. ameloprasum* var. *porrum*).

The genus *Allium*, included in the family *Amarylliaceae*, comprises a large number of species. Four of these species are well known by most people since they are food widely used in human nutrition: onion (*Allium cepa*), garlic (*A. sativum*), leek (*A. ameloprasum* var. *porrum*) and chive (*A. schoenoprasum*). Nevertheless, there are also many other wild varieties such as three-cornered leek (*A. triquetrum*), wild garlic or crow garlic (*A. vineale*), round-headed leek (*A. sphaerocephalon*), ramsons or bear’s garlic (*A. ursinum*), rosy garlic (*A. roseum*) and pale garlic (*A. paniculatum*), that are commonly found in the Mediterranean area [12,72] (Figure 2).

An infallible characteristic easily distinguishes plants of the genus *Allium*, which smell like garlic. However, this genus also presents other features, which are a basal bulb and sessile leaves without peduncle. The leaves have parallel veins and are concentrated in a basal rosette. At the end of winter or during spring, the plant is characterised by a long peduncle with a simple umbel of flowers with six equal perianth pieces [12].

The oxidative toxicity that onions and garlic cause to the erythrocytes is well known [73]. The toxicity is due to some organosulfur compounds present in all these plants. These compounds can damage the erythrocytes resulting in haemolytic anaemia with the formation of eccentrocytes and Heinz bodies, both of which result from oxidant injury to the membrane [72,74,75].

The *Allium* spp. toxicity is attributed to their content of disulfides, n-propyl disulfate and S-propyl cysteines sulfoxides (SMCOs) [12,72,74]. However, other researchers have identified at least five organosulfur compounds with potential oxidative damage to the erythrocytes of dogs (*Canis lupus familiaris*) [73]. The toxicity of these compounds is increased by the action of anaerobic bacteria that hydrolyse SMCOs to thiosulfonate and furtherly to dypropyl disulfides and dipropenyl disulfides. This pathway is especially important in ruminants [76]. The primary toxicological mechanism of *Allium* organosulfur compounds is oxidative haemolysis, which happens when oxidants in the erythrocytes exceed the capacity of the antioxidant metabolic pathways [72]. These compounds may cause a marked decrease in the activity of glucose-6-phosphate dehydrogenase (G6PD), causing an increase in methaemoglobin concentration, Heinz bodies count and reduce glutathione concentration in the erythrocyte. When the activity of G6PD decreases, glutathione concentration also decreases. This pathway causes an increase in hydrogen peroxide levels, causing the denaturation of haemoglobin by oxidising the sulfhydryl groups of the haemoglobin [77]. Simultaneously, sulfhaemoglobin formation occurs, and both compounds, denatured haemoglobin and sulfhaemoglobin, are less soluble than normal haemoglobin, causing precipitation and formation of Heinz bodies and eccentrocytes [72,75]. Heinz bodies have decreased deformability and are more fragile. They can burst when passing through sinusoids or small capillaries, causing intravascular haemolysis [78]. In addition, both the formation of Heinz bodies and eccentrocytes and the oxidative damage in the erythrocytes lead to extravascular haemolysis due to the activity of the reticuloendothelial system removing damaged erythrocytes [79,80].

Onion toxicosis has been widely described in a variety of domestic animals such as cats (*Felis catus*) [81], dogs [82,83], horses [84], cattle [85,86,87,88], sheep and goats [89,90,91] and even in water buffaloes (*Bubalus bubalis*) [92,93]. However, not all species show the same susceptibility. Cats, dogs and cattle appear most susceptible to the onion’s toxic effects, whereas sheep and goats are more resistant. The variety in susceptibility may be due to differences in haemoglobin structure and the detoxification pathways [94]. For example, catalase antioxidant activity in dogs is very low, while cats’ haemoglobin is about two to three times more susceptible to oxidative damage than other species. Both conditions may lead to an increased risk of *Allium* spp. toxicosis [95,96]. Sheep can be maintained on diets of up to 50% onions with no detrimental effects on their health [97], while diets exceeding 25% of dry matter in cattle are considered toxic [98].

Clinical signs are characterised by weakness, loss of appetite, ataxia, lethargy, tachypnea or tachycardia, dark red urine (haemoglobinuria) and pale or icteric mucous membranes [12,86,87,88]. A characteristic smell of onion or garlic is often detected when breathing [12,88]. Clinical signs may appear abruptly in the first 24 h after ingestion or, more frequently, become evident within days or weeks [12].

Diagnosis is based on clinical signs and knowledge of *Allium* spp. intake. The diagnosis should be supported by a blood smear, where it is easy to detect an increased Heinz body concentration in the erythrocytes. A complete urinalysis should also be performed to differentiate the origin of the red urine between haematuria and haemoglobinuria [12,88,99]. Urine test strips cannot distinguish, so a urine smear is recommended to detect the presence or not of erythrocytes. At necropsy, an intense garlic smell and paleness of icteric mucous membranes are observed. Histopathological study shows a centrilobular hepatic necrosis and degeneration and necrosis of tubular epithelial cells of the kidney. In addition, haemosiderin deposits in the liver, spleen and kidneys can be observed [12,88,93].

There is no specific treatment for onion toxicosis. However, some recommended treatments improve clinical signs and promote the recovery of affected animals. Firstly, it is encouraged to remove the animals from the area where they have fed the *Allium* spp. plants, or to restrict access [12]. Fluid or blood transfusion is recommended in severely affected animals [12,87,100]. Symptomatic treatment based on Vitamin E and selenium (Se) could be helpful due to their antioxidant effect. Phosphorus, Vitamin B complex and C and the supply of glucose or dextrose may improve the recovery of animals [93,99,100].

### 3.2. Brassicaceae Family

Another plant poisoning undergoing haemolytic condition is caused by the ingestion of plants of the family *Brassicaceae*. The *Brassicaceae* plants are generically referred to as *Cruciferae*. This family is vast and is a monophyletic group of about 338 genera and some 3709 species distributed worldwide [101]. The enormous number of species makes it impossible for all of them to share the same common characteristics. However, the best way to identify them is through the flower. The *Brassicaceae* have flowers with four petals and four sepals arranged in the shape of a cross (hence they are also named cruciferous). These flowers are mostly yellow and white; less frequently, they can be purple or pink. The flowers also have six stamens and are generally distributed in clusters [12].

**Figure 3:**
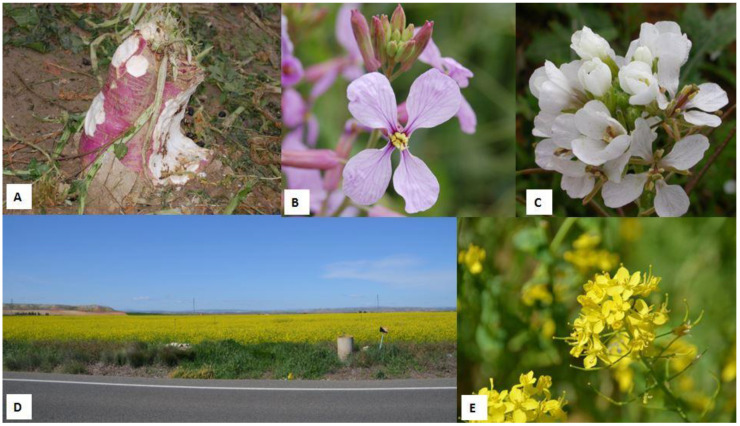
Some plants of the family *Brasicaceae*. (**A**) Turnip (*Brassica rapa* L. subsp. *rapa*) grazed by sheep as evidenced by the presence of faeces. (**B**) Detail of the flower of purple mistress (*Moricandia arvensis*). (**C**) Detail of the flower of white rocket (*Diplotaxis erucoides*). (**D**) A typical image of a canola/rapeseed (*Brassica napus*) field. (**E**) Detail of a cluster of flowers of canola plant (*B. napus*).

In this large family, there are many domestic and wild plants. Some of these plants are of great interest in human and animal nutrition. *Brassica* spp. is grown as forage crops for grazing in some countries to ensure the availability of fresh forage during shortage periods, mainly in summer and winter [102,103]. Additionally, these plants can be considered vegetable wastes of human nutrition and studies have determined that it is an excellent option to supply them to animals [104]. Some of these plants with crop interest are *Brassica oleracea* (kale, cabbage, brussels sprouts, cauliflower, broccoli, etc.), the radish (*Raphanus sativus*), some species of *Brassica rapa* (turnip, swede and rutabaga) and even species of *Brassica napus*, rapeseed being of particular interest due to the expansion of its cultivation during the last years. Another group of interest in this family consists of wild-growing plants that can be consumed as grass by livestock. The genera *Sinapsis*, *Diplotaxis*, *Capsella*, *Eruca*, *Sisymbrium* and some species of *Brassica* and *Raphanus* are included in this group [12,104] (Figure 3).

Some plants of this family are considered to have high forage quality, being a good source of high-level protein and digestible carbohydrates, especially leafy brasiccas such as kale (*Brassica oleracea* var *sabellica*). In addition, they are low in fibre, containing some bioactive compounds that might modulate ruminal fermentation and reduce CH4 emissions [103,105]. However, the chronic consumption of these plants may lead to some harmful effects due to the presence of some antinutritive factors. This issue poses the question of their feeding value in relation to their safety for ruminants [106]. Although the domesticated species grow on pastures, wild species tend to develop easily on embankments and fallow land, being the first plants to flower in late winter [12].

A brassica plant causing anaemia in cattle was first described by Rosenberg in 1939 in Germany [107]. However, a wide number of diseases and syndromes affecting cattle and other ruminants have been associated with brassica feeding. Some of these include haemolytic anaemia [103,106,107], goitre [12], nitrate toxicity [107], photosensitisation [108], rumen indigestion and acidosis [109] and also deficiencies of phosphorus and copper [110,111].

Occasionally, severe outbreaks are reported. Although initially the disease was known as “kale anaemia”, other species have demonstrated the same potential harmful effects, such as rape, turnips, brussels sprouts, etc. *Brassicaceae* species contain at least two crucial bioactive chemicals: glucosinolates and S-methyl cysteine sulfoxide (SmCo) [12,112,113,114]. Glucosinolates’ primary role is to defend the plant from harm, such as ingestion by livestock. After that, enzymatic hydrolysis of glucosinolates happened, releasing the sulphur compounds thiocyanates and isothiocyanates [114]. Initially, these derived compounds were associated with the clinical signs of anaemia observed after ingestion of brassica crops. However, these compounds are currently more associated with goiter [12,114].

On the contrary, the SmCo has always been associated with haemolytic anaemia since its detection in *Brassicaceae* plants [112]. Ruminant bacteria metabolise s-methyl cysteine sulfoxide in dimethyl disulphide, the active haemolytic agent, a compound similar to n-propyl disulfate of the *Allium* genus [12,113]. These compounds can affect detoxification pathways by disrupting thiol-containing enzymes such as glutathione reductase, reducing glutathione concentration in the erythrocyte. The reduction in antioxidant compounds and increased free radicals lead to erythrocyte membrane alteration, causing intracellular disruption and the haemolytic signs associated with this poisoning [113]. Thiocyanates derived from glucosinolates can also oxidise erythrocyte haemoglobin, causing the formation of Heinz bodies [115].

Animals affected by haemolytic anaemia show a lack of appetite, lethargy, tachycardia and tachypnea, dark red urine (haemoglobinuria) and pale or icteric mucous membranes. On rare occasions, the death of an animal may occur [12,102,116]. Other clinical signs may be observed if brassica poisoning results in goitre, photosensitisation or bloating [12,103,116] (Table 1).

The diagnosis is mainly based on clinical signs and knowledge of the consumption of these plants. It also can be supported by urine samples (dark red colour with high levels of haemoglobin) and blood smears (presence of Heinz bodies in erythrocytes). If any animal dies, the gross necropsy findings would be pale mucous membranes and congestion of the kidneys, liver and spleen. Histologically, zones of centrilobular hepatic necrosis and necrosis of tubular epithelial cells of the kidney can be seen. Widespread liver, spleen and kidney haemosiderosis is also frequently observed [12,116].

The treatment of the animals is based on supportive therapeutic measures based on fluid therapy and supplementation with vitamins and minerals. Vitamin E and selenium (Se) can be beneficial due to their antioxidant effect. Likewise, the supplementation of iodine to avoid goitre is recommended [12]. However, prevention should be the critical point in avoiding haemolytic anaemia disease. Ruminants should be introduced gradually to the brassica crop and allowed only limited daily access (estimated 70% or less of total diet dry matter). A companion crop of hay or cereals should be used to support the deficit [103,116]. Fortunately, Brassica-associated anaemia has dropped in the past decades. This is due mainly to the creation and cultivation of new cultivars that have significantly reduced the concentration of SmCo present in *Brassicaceae* species employed for cattle fodder [113].

**Table 1:** Clinical signs, toxic compounds and type of anaemia associated to each plant poisoning.

Plant	Toxic Compound	Type of Anaemia	Clinical Signs
Giant fennel (*Ferula communis*)	Ferulenol and ferprenin (coumarin derivatives)	Haemorrhagic anaemia	Bleeding from the nostrils. Blood in stools. Pale mucous membranes. Muscle weakness. Shallow breathing.
Bracken fern (*Pteridium aquilinum*)	Ptaquiloside (PQT)	Haemorrhagic anaemia	Severe thrombocytopenia. Hyperthermia and inappetence. Weakness. Pale and multifocal haemorrhages in mucous membranes. Epistaxis, haematuria and melena.
Sweet clover (genus *Melilotus*).	Dicoumarol (coumarin derivative)	Haemorrhagic anaemia	Lame due to muscle haemorrhages. Epistaxis, haematuria and melena. Pale mucous membranes. Subcutaneous haematomas. Depression.
*Allium* spp. pl	Organosulfur compounds	Haemolytic anaemia	Weakness and ataxia. Loss of appetite. Haemoglobinuria. Pale or icteric mucous membranes. Onion smell in breathe. Heinz bodies in blood smears.
*Brassicaceae* family	Organosulfur compounds.	Haemolytic anaemia	Lack of appetite. Hemoglobinuria. Pale or icteric mucous membranes. Tachycardia and tachypnea. Heinz bodies in blood smears.

## 4. Conclusions

Plant toxicology is a topic of great relevance in veterinary medicine, especially in herbivorous such as ruminants.

Anaemia caused by plant consumption should be considered while confronting anaemia signs in a herd. Although, in most cases, chronic consumption is needed, it should be avoided for feed animals over a potentially toxic dose. The most problematic moment is in drought or food scarcity periods, although the overgrazing of pastures could lead to poisoning in the most susceptible animals of the herd.

Fortunately, the increase in knowledge of these risks for livestock as well as the development of new forage varieties with less toxicity have reduced the appearance of these intoxications in recent years. In addition, the improvement in agricultural and forage conservation techniques has helped to improve the situation.

## Data Availability

Not applicable.
